# Supplementary material for: Are carbon emissions trading and green financial instruments synergistic? -Comprehensive quantitative research based on content analysis
Source: PLoS One. 2024 Mar 7;19(3):e0298601. doi: 10.1371/journal.pone.0298601 (PMC10919601; doi:10.1371/journal.pone.0298601)
Supplement: S1 Data — (ZIP) [file pone.0298601.s001.zip › Data/地级市Z_F工作报告.zip!/╔╜╢1⁄2/┴─│╟╩╨/└┤╫╘╣1⁄2╓┌║┼í╛┬φ┐╦╩2╛▌═°í┐_2011.docx]

各位代表：

　　现在，我代表市人民政府向大会报告政府工作，请予审议，并请市政协各位委员和其他列席的同志提出意见。

　　一、“十一五”时期政府工作回顾

　 “十一五”时期是我市发展历程中极不平凡的五年。五年来，面对国内外环境的复杂变化和重大风险挑战，我们坚持以邓小平理论和“三个代表”重要思想为指导，深入贯彻落实科学发展观，认真执行中央、省委省政府和市委的决策部署，积极作为、科学务实，迎难而上、奋力开拓，全市经济建设、政治建设、文化建设、社会建设和生态文明建设都取得巨大成就、登上新的台阶。全市粮食总产突破百亿斤大关，有色金属产业产值近千亿元，经济总量实现翻番，居民收入大幅跃升，成功创建国家卫生城市、环保模范城市，“十一五”规划确定的目标任务圆满完成！

　——综合实力迈上大的台阶。全市经济保持平稳较快发展，主要经济指标增幅高于全省平均水平。2010年全市生产总值达到1606.5亿元，是2005年的2倍，年均增长14.6%，人均生产总值由2005年的1480美元提高到4192美元；地方财政收入70.5亿元，是2005年的2.7倍，年均增长22%；社会消费品零售总额536.8亿元，是2005年的2.3倍，年均增长18.1%。各项存款余额1237亿元，贷款余额964亿元，分别是2005年的2.34倍、 2.32倍。茌平在山东西部首个跨入全国百强县。

　　——城乡面貌发生巨大变化。全市城市化水平由2005年31.8%提高到37.5%。聊城城区“一城五区”框架初步形成，城市功能显著增强，“江北水城·运河古都”品牌影响力进一步提高。各县（市）城区和小城镇面貌明显改观。农村“路、水、电、气、医、学”建设进一步加强，新居建设和危房改造全面展开。

——改革开放迈出重大步伐。农村经济体制、国有企业、财税体制、政府机构、事业单位改革有序推进，医药卫生体制改革全面启动。累计完成进出口总额115亿美元，为“十五”期间的6倍；利用外商直接投资5.1亿美元；招商引资到位资金897.3亿元，为“十五”期间的2.3倍。境外投资、境内外上市取得新突破。

　　——人民生活水平显著提高。全市城镇居民人均可支配收入达到17889元，农民人均纯收入6377元，分别是2005年的2倍、1.8倍，年均增长14.7%、13.1%。百人汽车拥有量由2005年的1.3辆提高到4.3辆。五年城镇新增就业45.5万人，城乡医疗卫生条件明显改善，覆盖城乡的社会保障体系逐步完善，各项社会事业全面发展，社会保持和谐稳定。

　　各位代表，五年来，我们重点抓了七个方面的工作：

　　（一）加大优质高效投入，经济发展后劲显著增强。我们坚持把抓投入、抓项目作为发展的关键，千方百计加大投入，坚定不移地上大项目、上好项目。坚决贯彻中央宏观调控政策，抢抓国家实施刺激经济增长一揽子计划等机遇，积极争取项目资金。应对国际金融危机以来，我市项目和资金均达到全省平均数以上，一举扭转了以往被动局面。2010年共争取项目资金82.1亿元，增长28.8%。坚持每年抓好100个重点项目，累计完成投资1174亿元，信发集团氧化铝粉、祥光铜业20万吨阴极铜、鲁西化工基地、中华电力2×60万千瓦机组、京九铁路聊城段电气化改造等一批大项目陆续完工、发挥效益；聊城火车站改造、邯济铁路复线、临清至高唐高速公路、济聊一级公路等正在建设，德商高速公路即将动工。“十一五”期间，全社会固定资产投资累计完成2879亿元，是“十五”期间的2.8倍，其中2010年完成887.7亿元，比上年增长22.5%。

　　（二）大力推进结构调整，产业发展水平明显提升。我们坚持把经济结构调整作为主攻方向，努力扩张总量、提升存量、优化结构、塑造特色，提升经济素质和产业竞争力。一是推进农业调整振兴。坚持“三农稳市”，全面落实各项支农惠农政策，2010年全市用于“三农”的支出占到财政总支出的34.9%。率先提出实施了《农业产业调整振兴计划》，突出抓好100个农业重点项目建设，大力实施“四百工程”。2010年，粮食生产实现连续八年丰收，总产达到104亿斤，突破100亿斤大关，标志着我市农业综合生产能力迈上一个新的台阶；蔬菜和食用菌、畜牧和水产、林果生产及加工产值分别达到142亿元、103亿元和102.6亿元。五年新增省级农业龙头企业22家，农民专业合作社发展到2988个；新增“三品”认证品种65个、基地面积137万亩。二是推进工业发展提升。坚持工业强市，努力加大工业投入，推进“五大基地”建设。我们制定了工业十大产业调整振兴规划，建立了战略性支柱产业推进落实机制，突出抓了有色金属、新能源汽车等优势产业的发展，我市被命名为“中国有色金属新城”，成为中国五大生产基地之一。全市规模以上工业增加值、利税、利润年均分别增长20.1%、33.1%和35.5%；利税过千万元的企业达到520户，其中过亿元的30户。三是推进服务业跨越发展。坚持三产兴市，制定了一系列得力措施，大力发展商贸物流、文化旅游，积极推进企业主辅分离。2010年，全市旅游总收入达到51.3亿元，年均增长18.6%；服务业增加值453.1亿元，年均增长17.4%。

（三）强化科技创新节能减排，转变发展方式取得新的成效。我们坚持把加快转变经济发展方式作为重大战略任务，进一步加大了科技创新、节能减排工作力度。一是大力推进科技创新。突出抓了技术创新平台建设和对外科技合作，全市新增国家级工程技术研究中心1家、省级17家，新增国家级技术中心5家、省级23家；我市被认定为有色金属材料及制品国家火炬计划特色产业基地、国家知识产权试点城市，高新技术创业服务中心被认定为国家级创业服务中心；与西安交大、省科学院等建立起全面战略合作关系，西安交大聊城科技园正在建设，快速制造国家工程技术中心在我市设立了示范中心。全市共获国家科技进步二等奖2项，省科技进步一等奖3项；2010年授权国内专利1262件，是2005年的6.03倍；高新技术产业产值比重达到28.6%，5年提高10.9个百分点。二是扎实做好节能减排。坚定果断地淘汰落后产能，突出抓好重点领域、重点企业节能减排工作，全市万元生产总值能耗、二氧化硫和化学需氧量排放削减量均超额完成任务；代表山东省参加全国海河流域水污染防治工作考核，取得第一名的优异成绩。2010年，我们又成功创建国家环保模范城，成为环保部采用新标准后第一个通过验收的地级市。可以说，我们在节能减排上打了一场漂亮的翻身仗！

　　（四）统筹抓好城乡建设，全市面貌发生巨大变化。我们坚持把加快城镇化作为重大战略措施来抓，启动和实施了“三个百亿元工程”。坚持城建靓市， 五年累计完成城市建设开发投资568亿元，其中2010年达到241.6亿元，是上年的4.7倍。按照“江北水城·运河古都”的战略定位，进一步加大了聊城城区规划建设工作力度，先后实施了一系列城建重点项目并取得重大进展，市城区高层建筑、标志性建筑快速增加，大城市特色和氛围逐步形成；古城保护与改造进展顺利，中华水上古城风貌初步彰显；东昌湖风景区进一步完善，被评为国家“4A”级风景区；实施了运河四期开发，形成了蜿蜒8公里的古运河风光带；旅游接待中心、体育公园主场馆、铃铛湖、状元岛等重点工程全面完成。一批设计漂亮新颖、居住宽敞适用的新型居民小区交付使用，居民居住环境有了新的改善。积极推进“五城同创”，继国家优秀旅游城、双拥模范城之后，又成功创建国家卫生城、国家环保模范城，创建国家园林城工作取得阶段性成果，2008、2009年度分别被评为中国“十大特色休闲城市”、“十大宜居城市”。各县（市）城区和小城镇建设取得显著成效。五年全市城镇新增道路面积579万平方米，集中供热面积618.6万平方米，用水普及率达到97.5%；八县（市、区）城区全部通上管道天然气，在全省第一个实现“气化山东”目标；新建污水处理厂11座、垃圾处理厂3座，污水处理率达到95.2%。坚持统筹城乡发展，加强新农村建设，新建农村公路6470公里、客运站108个，改造农村危桥1311座，所有行政村实现了通油路、通客车。新建沼气池12.7万个、太阳能热水器14.2万平方米，新增315万人用上自来水、33万户看上有线电视，农民生活条件明显改善。积极推进农村新居建设和危房改造，启动千户社区140个、城中村改造86个、整村改造356个；建成农村新居18.4万户，改建危房3.5万户，先后有30多万农民群众告别了低矮落后的旧式住房，搬进了宽敞、明亮、舒适的新式楼房。可以说，过去的五年是我市城乡面貌变化最快，特别是城乡居民居住条件改善最快的五年！

　　（五）深入推进改革开放，经济发展活力进一步增强。我们坚持把改革开放作为经济社会发展的强大动力，坚定不移地加以推进。国有资产监管体系不断完善，企业改革继续深化。继中通客车、东阿阿胶、鲁西化工上市之后，再次实现鲁西化工定向增发，华泰化工、纷美包装分别在创业板和香港上市。农村综合改革稳步推进，第二轮土地承包政策全面落实，农民承包土地流转43.7万亩；集体林权制度改革全面推开，主体改革任务基本完成。民营经济保持快速发展，占全市生产总值比重达到51%。市、县两级政府机构改革基本完成。医药卫生体制改革稳步推进。积极实施“走出去、请进来”战略，举办了2010海内外知名企业家齐鲁行等一系列招商活动，与华润、中色、中冶、中盐、中粮、大通等知名央企成功实现对接，借力发展初见成效；信发集团、祥光铜业等先后在国外建立了原料基地，冠丰种业巴西棕榈系列开发成为全省最大的农业对外投资项目，全市境外投资企业达到40余家，协议投资额2.8亿美元。国际友好城市达到3个。

　　（六）着力保障改善民生，社会事业全面进步。我们坚持把改善民生作为政府工作的出发点和落脚点，在财政比较困难的情况下，加大投入，加快发展。全市财政用于民生的比重逐年增加，2010年达到84.2亿元，占到财政总支出的58.1%。就业工作成效明显，城镇登记失业率为3.33%；新增农村劳动力转移就业52.3万人次。城镇居民基本医疗保险全面推开，各类保险覆盖面进一步扩大；农村新型合作医疗参合率达到99.7%，新型农村社会养老保险试点进展顺利，五保对象集中供养率达到72%；城市低保标准最低提高420元，农村提高400元。城市住房保障力度加大，新增廉租住房补贴4357户，建设经济适用房9482套，完成棚户区改造3462户，启动了公共租赁房建设试点。各级各类教育均衡协调发展，新建农村中小学校舍51.7万平方米，对义务教育阶段学生免除了杂费、农村学生免除了课本费，高中段入学率达到86.7%，素质教育取得阶段性成果；市直职业学校资源整合实现突破，组建了聊城高级财经职业学校和高级工程职业学校。卫生事业发展加快，在全省率先实施了“农民健康服务行动”，22.5万最偏僻乡村的农民群众不用出门就享受到全市最优质的医疗服务；新建改建城市社区卫生服务中心16处、社区卫生服务站94处，完成了81个乡镇中心卫生院、1500个村卫生室的建设或改造；甲型H1N1流感、手足口病等传染性疫情得到有效控制。文化事业全面进步，文化信息资源共享工程、农村公益电影放映工程全面完成，中国运河文化博物馆完成布展并免费开放，运河申遗取得阶段性成果。体育工作取得新成绩，圆满完成了第十一届全运会聊城分赛区办赛工作，并取得2金、1银、1铜的历史最好成绩，群众性体育活动更加广泛深入。人口与计划生育工作进一步加强，人口自然增长率为5.7‰，第六次人口普查基本完成。全力做好支援四川灾区工作，各项援建任务圆满完成。深入开展“平安聊城”建设，食品药品监管取得明显成效，安全生产形势保持稳定，平安奥运、平安世博、平安全运等重大安保任务全面完成。新闻出版、社会科学、妇女儿童、青少年、慈善、残疾人、老龄事业持续发展，档案、史志、气象、防震减灾、油区等工作稳步推进，外事侨务、对台工作取得新的成绩。

　　（七）切实加强自身建设，服务水平明显提升。我们坚持把加强自身建设作为做好政府工作的根本保证，努力提升服务水平。深化行政审批制度改革，精减各种审批、收费233项；市行政服务中心作为全国唯一标准化试点，顺利通过国家验收。实施了政府部门目标责任管理，强化行政问责，政府工作效能明显提高。出台了政府依法行政企业评价制度和“八条禁令”，严肃查处十三种行政不作为、乱作为行为，发展环境明显改善。积极推进政务公开，市政府所有部门实现了网上信息公开，市长公开电话承办事项办结率达到98.6%。修订了《聊城市人民政府工作规则》，进一步推进政府工作规范化、制度化。加强财政资金管理，开展了“小金库”集中清理，规范了规费减免程序，对公款出国旅游进行了专项治理。加强政府应急管理工作，应对突发事件的能力明显增强。切实加强廉政建设和反腐败斗争，实行了市直纪检监察派驻机构统一管理，严肃查处了一批违法违纪案件。

　　各位代表，五年来，国际国内形势跌宕起伏、复杂多变，特别是国际金融危机对我市经济社会发展造成严重冲击。面对复杂形势和前所未有的困难和挑战，我们能够取得这样的成绩是非常不容易的。这得益于中央、省委省政府和市委的科学决策、正确领导，得益于各级各部门齐心协力、扎实工作，得益于人大、政协和社会各界加强监督、大力支持，更是全市人民聪明才智、辛勤劳动的结晶！在此，我代表市人民政府向全市工人、农民、知识分子，向所有企业家和纳税人，向各民主党派、工商联、各人民团体和各界人士，向驻聊人民解放军、武警官兵、公安干警和中央、省驻聊单位，向关心和支持聊城发展的海内外朋友，表示衷心的感谢和崇高的敬意！

　　各位代表，五年取得的成绩来之不易，积累的经验弥足珍贵，创造的精神财富影响深远。一是坚持解放思想、更新观念，不断创新工作思路。深入开展“解放思想大讨论”活动，确立了山东东引西拓“桥头堡”、“一五二”基地的发展目标；根据形势要求，组织开展了“谋划年”、“落实年”、“项目突破年”、“全面提升年”活动，有力地促进了经济社会又好又快发展。二是坚持抢抓机遇、危中求机，用足用好各种有利条件。面对国际金融危机的严峻挑战，坚持“化危为机、弯道超越”，开展“项目突破年”活动，实施产业提升工程，项目和资金争取、重点项目建设实现新的突破。三是坚持科学发展、和谐发展，努力提升发展水平。提出了建设生态文明市的任务和生态型强市名城的奋斗目标，大力推进结构调整和发展方式转变，可持续发展能力明显增强。四是坚持深化改革、扩大开放，不断增强发展活力。大力推进各项改革，努力扩大对内对外开放，为经济社会发展提供了强大动力。五是坚持立足当前、着眼长远，努力为未来发展打牢基础。制定了一系列经济发展规划，大力培植战略性支柱产业、新兴产业，推进重大交通基础设施建设，在保持当前经济平稳较快发展的同时，为未来的跨越发展奠定了良好基础。六是坚持以人为本、关注民生，让广大群众共享发展成果。切实加大民生投入，加快发展各项社会事业，及时解决经济社会发展中的突出矛盾和问题，人民群众得到了更多实惠！

各位代表，在充分肯定成绩的同时，我们也清醒地看到，我市经济社会发展仍然存在一些矛盾和问题。主要是：经济总量小，人均占有水平低，新的经济增长点还不够多；产业结构不合理，工业产业链条较短，新兴产业规模较小，服务业发展相对滞后；自主创新能力不强，高技能、高层次人才匮乏；资源消耗型企业、重污染企业较多，资源、环境压力较大；社会事业和社会保障还难以满足群众的实际需要，安全生产、社会稳定领域的问题仍时有发生；少部分政府工作人员开拓创新意识不强、办事效率不高，发展环境还存在一些问题。对这些矛盾和问题，我们一定要高度重视，采取更加有效的措施加以解决。

　　二、“十二五”时期经济社会发展的总体要求

　　“十二五”时期是我市全面建设小康社会、加快生态型强市名城建设实现新跨越的关键时期，是深化改革开放、加快经济发展方式转变的攻坚时期。制定并实施好“十二五”规划，具有重要的现实意义和深远的历史意义。对“十二五”规划编制工作，市委、市政府高度重视，2009年底就作了全面安排部署。编制工作启动之初，组织有关部门和各方面专家，对经济社会发展14个重大问题作了专题研究。编制过程中，聘请国家发改委、省发改委有关领导，中国工程院院士，北京交通大学教授等组成专家组进行咨询论证，在全市开展了“我为‘十二五’规划建言献策”活动。最近召开的中共聊城市委十一届八次会议通过了《关于制定国民经济和社会发展第十二个五年规划的建议》。在以上基础上，我们制定了《国民经济和社会发展第十二个五年规划纲要（草案）》，已印发大会，请一并予以审议。

　　“十二五”时期，国际国内形势将继续发生深刻变化。从国际看，世界经济有望在调整中逐步恢复增长，但不稳定不确定因素仍然较多；世界经济结构进入调整期，创新和产业转型处于孕育期，世界经济格局正在发生复杂深刻变化。从国内看，我国仍处于重要战略机遇期，各种有利条件依然存在，但也面临不少矛盾和问题；中央提出以科学发展为主题，以加快转变经济发展方式为主线，将进一步加大经济结构战略性调整、科技进步和创新、保障和改善民生、节约资源和环境保护等项工作力度。从全省看，省委、省政府加快推进山东半岛蓝色经济区、黄河三角洲高效生态经济区、省会城市群经济圈等重点区域发展，以实施重点带动战略为抓手，促进区域经济协调发展；周边各市竞相发展，形势逼人，区域间竞争更加激烈。面对难得的历史机遇，面对诸多风险和挑战，我们必须树雄心、立壮志，奋发有为、奋力开拓，使经济社会发展的更快、更好，推动聊城跨越发展。这是时代的要求，人民的意愿，是历史赋予我们的庄严使命，我们一定要承担起这个历史责任！

　　根据党的十七届五中全会精神，结合我市实际，“十二五”时期经济社会发展的总体要求是：坚持以邓小平理论和“三个代表”重要思想为指导，深入贯彻落实科学发展观，以科学发展为主题，以加快转变经济发展方式为主线，以改革、开放、创新为动力，加快推进工业化和城镇化进程，加快经济结构战略性调整，着力打造“一五二”产业基地和山东省东引西拓“桥头堡”，努力“再创新优势，实现新跨越”，为全面建成小康社会、实现生态型强市名城目标打下坚实基础。主要目标是：全市生产总值年均增长13%，三次产业比重大体调整为10∶54∶36，地方财政收入年均增长18%，全社会固定资产投资年均增长18%，社会消费品零售总额年均增长17%，城镇居民人均可支配收入年均增长10%以上，农民人均纯收入年均增长13%，确保完成省下达的节能减排约束性目标。

　　“十二五”时期经济社会发展目标的确定，主要把握了三个方面的原则：一是坚持科学发展。要以科学发展为主题，更加注重经济增长的质量和效益，更加注重全面协调可持续发展，更加注重统筹兼顾，既要实现经济社会又好又快发展，又要为长远发展打牢基础。二是坚持争先进位。要立足跨越发展，把发展作为解决所有问题的关键，扭住发展不放松，加快发展不动摇，确保在提高发展质量和效益的前提下，主要经济指标增幅不低于全省平均水平，不低于周边市水平，力争位次前移。三是坚持民生优先。要坚持以人为本、共享发展，在经济发展的同时，更加注重改善民生，更加注重社会公平，努力实现居民收入增长和经济发展同步、劳动报酬增长和劳动生产率提高同步，使发展成果惠及广大人民群众。

各位代表，“十二五”期间，我们要在现有基础上，“再创新优势，实现新跨越”，努力打造山东西部新兴生态工业城市、冀鲁豫交界地区商贸物流中心城市、江北文化旅游和休闲度假目的地城市。立足长远发展，着眼提高聊城未来竞争力，我们要再创四大发展新优势：

一是再创产业发展新优势。以打造“一五二”基地为着力点，加大优质高效投入，努力扩总量、转方式、调结构、塑特色，建立起更加富有竞争力的现代产业体系，实现产业发展新提升。农业方面，围绕建设生态农业及农产品深加工基地，着力实施农业产业调整振兴计划，稳定粮棉生产，做大做强蔬菜、畜牧、林果等优势产业，建设全国知名的绿色有机农业示范区，加快由农业大市向农业强市的转变。工业方面，着力培植战略性支柱产业、培育战略性新兴产业、发展民营特色产业、壮大骨干龙头企业，加快建设有色金属及金属加工、运输设备及零部件、基础化工及精细化工、轻纺造纸及食品医药、能源电力及节能设备五大基地，形成一批新的优势产业、骨干企业。服务业方面，坚持扩大规模与提高水平并重，加快建设商贸流通及现代物流、文化旅游及休闲度假两大基地，着重发展一批龙头企业，形成服务业发展的新亮点。

二是再创基础设施新优势。立足当前，着眼长远，大力加强交通、能源等基础设施建设，努力再上新水平，为实现新跨越提供支撑。重点抓好一批重大铁路、公路项目和城际轨道交通、军民合用机场建设，实现聊城到各县（市）、聊城融入济南两个“半小时”经济圈， 8个县（市、区）全部通高速路，确立聊城全国重要区域交通枢纽地位。

三是再创体制机制新优势。坚持把改革作为强大动力，坚定推进经济、政治、社会、文化等领域改革，调动各方面改革积极性，尊重群众首创精神，加快形成有利于科学发展的体制机制，进一步增强经济社会发展的活力。

四是再创科技人才新优势。科技、人才是区域发展的核心竞争力所在。聊城发展的根本出路在于科技、人才。“十二五”期间，我们要大力实施技术创新工程，深入实施人才强市战略，着力在重点领域和关键技术开发利用、高端人才凝聚、高技能人才培养上实现突破，为经济社会跨越发展提供支撑。

到“十二五”末，经过全市上下的共同努力，我们要在五个方面实现新跨越。

一是实现综合实力新跨越。保持经济平稳较快发展，提高经济增长质量和效益，全市生产总值力争突破3000亿元，地方财政收入达到160亿元以上，全社会固定资产投资五年完成7200亿元。经济结构进一步优化，农业基础更加稳固，战略性支柱产业全面形成，战略性新兴产业形成规模，服务业实现跨越发展，经济综合竞争力显著增强。

二是实现城乡建设新跨越。大力推进城乡一体化，构建以市城区为龙头、县（市）城区和中心镇为主体、农村社区为基础的现代城镇体系，全市城镇化水平达到50%左右。按照“一城五区”的总体框架，推进中心城区东扩南展，全面抓好中心城区、东部经济开发区、北部物流园区、西部现代服务业示范区和南部城市新区建设，力争人口达到100万左右。抓好7县（市）城区建设，拉大城市框架，完善城市功能，临清、茌平、高唐城区人口达到30万左右，其他县达到20万左右。抓好30个中心镇建设，着力完善基础设施和公共服务，引导周边人口向中心镇集中。加快农村社区建设，建成1000个农村社区，逐步实现农村社区化。

三是实现对外开放新跨越。实施更加积极主动的开放战略，全面加强国际交流和区域合作，进一步提高外向型经济发展水平。外贸发展方式加快转变，2015年进出口总额达到70亿美元，年均增长15%。利用外资质量和水平大幅提升，累计达到7亿美元。“海外聊城”建设取得重大进展，重要资源境外供应基地基本建成，重点企业跨国并购取得突破。

四是实现民生改善新跨越。我们要更加关注社会建设和民生改善，做到民生投入比重再提高、公共服务覆盖面再扩大、民生建设再上新水平，努力形成与经济社会发展相适应的现代教育体系，建立起覆盖城乡居民的基本医疗卫生体系、社会保障体系。文化体育等各项社会事业全面发展，社会保持和谐稳定。

五是实现生态环境新跨越。我们要坚持经济发展与生态保护并重，牢固树立绿色、低碳发展理念，健全生态市建设长效机制，加强生态建设和环境保护，推进资源节约，发展循环经济，努力实现资源环境与经济社会协调发展。

各位代表，“十二五”规划目标宏伟，任务艰巨，前景美好。我们一定要苦干实干，用我们的辛勤劳动和汗水，把“十二五”规划的宏伟蓝图变为美好现实，使聊城成为全省经济发展最快、环境质量最优、群众得到实惠最多的城市之一！

　　三、2011年的目标任务和工作重点

　　2011年是“十二五”规划的开局之年，也是中国共产党成立90周年，做好今年的政府工作意义十分重大。我们要按照中央、省委省政府和市委的决策部署，按照“十二五”规划的目标任务，坚持积极作为、科学务实、争先进位、奋力赶超的工作基调，扎实开展“继续提升年”活动，着力在推进经济结构战略性调整、提高自主创新能力、强化节能减排和环境保护、深化改革开放、保障和改善民生上取得新的进展，促进经济平稳较快发展和社会和谐稳定，确保“十二五”开好局、起好步，以优异成绩迎接建党90周年。主要预期目标是：生产总值增长13%，地方财政收入增长18%，全社会固定资产投资增长20%，利用外商直接投资增长10%，外贸进出口增长15%，社会消费品零售总额增长17%，城镇居民人均可支配收入增长10%，农民人均纯收入增长13%，居民消费价格涨幅控制在4%左右，城镇登记失业率控制在4%以内，万元生产总值能耗等节能减排指标全面完成省下达任务，人口自然增长率控制在7.7‰以内。

　　工作中，着力抓好以下八个方面：

　　（一）切实扩大投资和消费，促进经济又好又快发展。抓住国家扩大内需的机遇，努力扩大投资和消费，增强对经济增长的拉动力。一是坚持不懈地扩大优质高效投入。继续抓好100个重点项目建设，计划完成投资482亿元。加强项目管理，规范建设手续，完善协调推进机制，加快项目建设进度。调整优化投资结构，加大战略性支柱产业、战略性新兴产业和服务业投入，支持节能环保、自主创新和技术改造，引导投资继续向“三农”、保障性安居工程、教育卫生等民生领域倾斜。积极拓宽融资渠道，加大上级扶持项目资金争取力度，加强银企、银地合作，推动优势企业、高科技中小企业直接融资，努力扩大民间投资，为加大投入提供资金支持。二是努力扩大消费需求。落实好国家收入分配改革政策，建立企业职工工资正常增长机制和支付保障机制，切实增加居民收入。积极落实扩大消费的信贷政策，继续实施家电下乡、家电以旧换新、建材下乡，发展新型消费业态，培育消费热点。整顿和规范市场秩序，加强消费者权益保护，营造有利于消费的政策环境。

　　（二）深入实施农业产业调整振兴计划，加快农业农村发展步伐。围绕建设生态农业及农产品深加工基地，持续推进农业产业调整振兴，设立农业产业调整振兴基金，以100个农业重点项目为抓手，以完善产业体系为重点，进一步加大农业投入，努力在以下六个方面实现突破：一是在现代农业示范发展上实现突破。抓好市级现代农业、莘县特色蔬菜、东阿优质粮食、东昌府区生态观光农业、黄河故道现代林业等现代农业示范园区建设，力争在全省率先建成现代农业示范市。二是在基地建设上实现突破。抓好食用菌、大蒜、黄瓜、西葫芦、茄子、西红柿、韭菜、油菜、西瓜、香瓜等“十大蔬菜基地”建设，积极参与沿黄肉牛肉羊、山东半岛高端禽肉、沿高速公路奶牛、鲁中南生猪等区域性基地建设，推进种养业规模化、区域化。三是在提高农业组织化程度上实现突破。继续扶持国家、省、市重点龙头企业发展，加大对成长型农产品加工企业扶持力度，争取新增规模以上农业龙头企业10家。积极发展农民专业合作社，加强政策扶持，规范运作机制，扩大经营规模。四是在提升农产品质量安全水平上实现突破。推进农业标准化生产，新增无公害农产品、绿色食品、有机食品认证20个，基地面积10万亩，打造一批具有聊城特色、在国内具有较高知名度的名牌农产品。五是在农产品产销对接上实现突破。加快聊城农产品批发大市场建设，新建改建农产品批发市场10处，逐步在全市形成功能完善、衔接顺畅的农产品批发市场体系。继续加大“农超对接”力度，加快聊城农产品网上市场建设。六是在水利基础设施建设上实现突破。抓住中央加大水利工程投入的机遇，实施位山、陶城铺、彭楼等灌区续建配套与节水改造，抓好茌新河等4条中小河流治理，新打机井2000眼。以沉沙池区、黄河故道为重点，水土保持综合治理面积30平方公里。各位代表，我们要以增加农民收入为核心目标，加快实施农业产业调整振兴计划，争取通过今明两年的努力，全市农民人均纯收入达到或接近全省平均水平！

　　（三）大力实施“4455”工程，营造工业发展新优势。围绕“五大基地”建设，持续推进工业做大做优，设立战略产业发展基金，推进“4455”工程实施。一是培植四大战略性支柱产业。大力培植铜及铜加工、铝及铝加工、新能源汽车和精细化工等四大战略性支柱产业，使其逐步成为支撑聊城发展的“千亿产业”。发挥各产业龙头企业的带动作用，以项目建设为抓手，加快信发循环经济示范园、祥光生态工业园、时风汽车产业园、中通新能源汽车产业园、鲁西化工循环经济产业园建设，推动产业规模迅速膨胀。坚持走高端高质高效发展路子，着力向精深加工延伸、向高端价值链突破，努力提高产业竞争能力。同时，按照建设“五大基地”的要求，抓好其他产业的发展。二是培育四大战略性新兴产业。围绕新能源、生物医药、节能环保和高端装备制造等新兴产业，找准发展突破口，迅速膨胀规模，尽快形成一批具有自主知识产权、掌握核心技术、市场竞争力强的重点企业，提升全市产业发展的层次和水平，高新技术产业产值占规模以上工业比重提高2个百分点以上。三是发展五大民营特色产业。实施特色产业提升计划，以轴承、冶金板材、钢管、钎具、畜禽加工等五大产业为重点，大力发展特色产业，培植产业集群，逐步形成各具特色、竞相发展的区域经济发展格局。四是壮大50户龙头骨干企业。实施大企业带动战略，重点抓好50家龙头骨干企业的发展，在资金、土地、项目申报等方面给予重点支持，促使企业夯实技术、质量、管理、品牌基石，由市内优势企业向省内、国内行业领军企业发展，在全市经济发展中发挥引领、带动作用。各位代表，落后只能代表我们的过去，绝不会再代表我们的未来，只要我们按照既定的工作思路和目标，坚持不懈地抓下去，聊城工业一定能够率先崛起，一定能够创造新的辉煌！

　　（四）突出抓好商贸物流、文化旅游，推动服务业跨越发展。围绕商贸物流及现代物流、文化旅游及休闲度假两大基地建设，持续推进服务业跨越发展，确保服务业增加值占生产总值比重提高2个百分点。一是大力发展商贸物流业。加快推进铁路“四路一站”、公路“四路一枢纽”、军民合用机场建设，打造区域交通枢纽。加快发展现代物流业，抓好聊城物流园区、公共物流信息中心、煤炭储运基地建设，积极引进国内外知名物流企业，打造区域性物流服务枢纽；全面推进企业主辅分离，使其向第三方物流发展。抓好城市中心商业区建设，以百货大楼、五星百货、银座商城为龙头，打造集零售、餐饮、娱乐、商务于一体，具有较强竞争力的核心商业集聚区。抓好香江、烟店轴承、大东钢管等专业批发市场发展，进一步提高对外影响力和辐射力。继续实施“万村千乡”市场工程，新建1000个标准化“农家店”、5个物流配送中心。鼓励发展连锁经营、特许经营、电子商务等现代流通方式，提高流通现代化水平。二是加快发展文化旅游业。围绕名胜古迹、温泉度假、阿胶养生、森林生态等旅游主题，抓好中华水上古城、徒骇河世界运河（建筑）博览园、马颊河生态旅游度假区、古漯河生态休闲观光走廊、临清中州古城、阳谷千年古城和蚩尤陵开发等重点项目建设。整合旅游资源，实施精品工程，抓好旅游线路的设计和营销。继续实施“五个一”精品工程，办好“五一”、“十一”特色旅游节庆活动。加快旅游集散中心建设，加大对济南都市圈、京津地区等重要旅游客源地的宣传推介力度，努力扩大客源市场。大力培育龙头文化企业、特色文化品牌和文化产业群，推动文化旅游互促互动。三是积极发展其它服务业。规范发展房地产业，加强面向低收入群体的中小户型、中低价位普通商品住宅建设。支持发展金融业，积极引进全国性、区域性银行业金融机构，推动农村信用社银行化改革，积极稳妥地发展村镇银行、农村资金互助社、小额贷款公司、融资性担保公司，规范发展各类保险机构。加快发展农村服务业，支持农业服务机构开展种子统供、重大病虫害统防统治等项服务。鼓励发展信息服务、社区服务、中介服务等服务业。

　　（五）全面加强城乡建设，加快新型城镇化进程。坚持城镇化、工业化统筹安排、协调推进，进一步加快新型城镇化、城乡一体化进程，城镇化水平力争提高2个百分点。

突出抓好城市建设。坚持完善城市功能、提升城市品位与扩大城市规模并举，抓好中心城市建设，为打造百万人口大城市创造条件。重点实施八大工程：一是古城保护与改造。完成西城门、北城门、瓮城及其部分城墙，建设南北大街，恢复部分历史景点，实施四个片区水系景观改造。二是徒骇河世界运河（建筑）博览园。实施城区段防洪治理，建设滨河大道，启动两岸开发。三是创建国家园林城市。实施绿化“增量升级”，突出抓好居住小区、单位庭院、公园和街旁绿地建设，提升园林绿化水平。四是湖河水系完善工程。完成运河五期、南关岛、名人岛建设，实施东南湖、班滑河整治。五是城中村改造。实施14个改造项目，完成70%以上改造任务。六是基础设施提升工程。新建改造湖滨路、利民西路等13条道路，建设东昌路跨路高架桥，完成火车站升级改造。新增集中供热面积100万平方米、管道燃气用户1万户。今明两年更新节能减排型公交车100部。七是文化体育工程。完成市民活动中心主体工程。加快体育公园建设，完成全民健身活动中心，启动主体育场、游泳馆、运动员公寓建设。启动全省青少年足球训练基地建设。八是环境整治工程。加强违规建筑、违规经营治理，推进沿街经营相对集中，进一步抓好环境卫生综合整治，提升城市管理水平。同时，抓好各县（市）城区和30个中心镇建设，努力增强对经济发展的承载力、对农村人口的吸纳力。

　　加快推进新农村建设。按照城乡一体化发展的要求，全面加强农村基础设施和环境建设，努力改善农村面貌。一是继续抓好农村交通建设。新建改建农村公路260公里、农村危桥135座，县乡公路综合完好路率达到75%以上。积极推进城乡客运公交一体化，使老百姓出行更加便利。二是大力推进农村新居建设和危房改造。坚持尊重农民意愿、维护农民利益，用足用好增减挂钩政策，投入资金50亿元，建设农村新居5万户，改造危房1.5万户，建设千户社区100个，合并村庄300个，配套实施农村道路、绿化、排水、污水处理、垃圾处理等基础设施。三是努力改善农民生活条件。解决10万人饮水安全问题，新增有线电视4万户，推广户用沼气池2万个、太阳能2万户。

　　积极推动农村人口向城镇转移。把中低价位住房建设作为加快城镇化进程的突破口和着力点，努力为农村人口向城镇转移创造条件。切实加大保障性住房建设力度，新建经济适用房1453套、廉租住房200套、公共租赁住房3000套，实施棚户区改造9410套，增加廉租住房补贴200户。面向新就业大学生、进城务工农民和城市中低收入家庭，重点加强限价房建设，促进人口向城镇流动，膨胀城市规模，加快城镇化进程。我们要真抓实干，不懈努力，争取在不远的将来，实现城市中低收入的家庭和人员住有所居、安居乐业！

　　（六）大力推进科技创新和节能减排，促进经济发展方式转变。继续把科技创新和节能减排作为加快转变经济发展方式的关键，进一步加大工作力度，确保取得新的成效。一是大力推进科技创新。加强高层次创新平台、优势产业公共技术平台建设，支持企业创建省级以上工程技术研究中心、技术中心。强化同高校院所的合作，抓好西安交大聊城科技园建设，推动产学研合作实体化。加强高新技术创业服务中心建设，进一步提升服务功能。按照国内领先、国际一流的要求，下大力推进九州国际高科园建设，努力打造支撑聊城未来跨越发展的高科技高地。继续推进信息化、工业化融合，加快信息技术推广应用。大力实施质量兴市战略和标准化发展战略，建设面向重点产业产品的国家级质检中心，培育和创建一批优质产品生产基地。二是切实抓好节能减排。加大重点行业、重点企业节能技术改造力度，推行合同能源管理，加快淘汰落后产能。积极开展清洁生产，加快主导产业循环经济模式示范推广。继续抓好环境保护，加快减排工程建设进度，加强流域污染、大气污染防治，推进垃圾分类处理，全市污水处理率达到85%以上，垃圾无害化处理率达到90%以上。加强生态保护，推动人工湿地建设，努力保持良好的生态环境。我们一定要在加快经济发展的同时，使聊城的天更蓝、地更绿、水更清、城更美，使人民群众生活得更开心、更幸福！

　　（七）继续深化改革扩大开放，为经济社会发展提供强大动力。全面推进各领域改革、拓展国际国内合作空间，提高对内对外开放水平，努力增强经济发展内在活力。

　　加大改革攻坚力度。积极推进农村改革，加大农村产权流转市场培育，鼓励农民流转土地承包经营权，全面推进集体林权制度改革。深化国有企业和国有资产管理体制改革，加快市属国有企业股份制改造，选择有条件的企业开展独立董事试点，加快困难企业退出步伐，力争企业上市取得新的突破。抓好财税改革，做好扩大增值税征收范围、财政省直管县等项工作。推进事业单位改革，规范完善公开招聘、人员退出等制度，推动经营性单位转企改制。

　　努力扩大对内对外开放。一是继续下大力开展招商引资。坚持创新招商模式、优化发展环境与强化目标责任考核相结合，以世界500强、大型央企等国内外知名企业为目标，以港、台、日、韩等国家和地区，以及国内的珠三角、长三角、京津、山东半岛蓝色区为重点，着力增强招商引资的针对性和实效性，努力扩大招商引资规模。二是积极调整外贸结构。引导企业加大研发投入，创建自主品牌，努力增加高科技含量、高附加值产品出口，增创外贸竞争新优势。调整出口市场结构，高端产品要重点开拓发达国家市场，实现效益最大化；中低端产品要积极开拓东盟、中东、非洲、拉美等新兴市场，提高市场占有率。三是大力实施“海外聊城”战略。以长远的、战略的眼光推进对外开放，围绕开发资源、开辟市场两大重点，突出抓好铝土矿、铜精矿、棕榈树和天然橡胶、驴养殖和加工、木材和木浆等五个境外资源供应基地建设，努力提高聊城未来发展所需稀缺资源的供应保障能力。支持信发集团、祥光铜业等重点企业在非洲、南美等地建立工业园区，抢抓先机，占领市场，扩大规模，提高效益。四是举全市之力加快经济开发区发展。进一步加大支持力度，着力增强内生动力，推动开发区跨越发展，努力成为创新能力强、财税贡献大、资源消耗少、环境质量优的高端产业聚集区，力争早日建成国家级开发区。

　　（八）完善基本公共服务，维护社会和谐稳定。我们要把不断提高人民群众的生活水平和质量作为一切工作的出发点和落脚点，在设立社会公益基金的同时，进一步加大对民生的投入力度，使人民群众得到更多的实惠和服务。

　　做好就业和社会保障工作。突出抓好高校毕业生、农民工和城镇困难人员三大群体就业工作，力争新增城镇就业6万人，其中失业人员再就业2万人，帮扶困难群体就业4000人。落实自主创业人员一次性岗位开发补贴和扶持资金，推进创业带动就业。抓好社会保险扩面征缴，重点抓好新增规模以上企业和灵活就业人员扩面工作，解决好历史遗留的关闭破产集体企业退休人员医疗保障和“老工伤”问题，城镇医疗保险参保率达到96%。推进社会保险关系跨地区转移接续，促进参保人员无障碍流动。进一步提高城市低保标准和生活补贴，农村低保标准提高到1400元。完善农村五保供养制度，提高生活补助标准，稳步推进东昌府区、茌平县新型农村社会养老保险试点工作。加大社会救助工作力度，重点抓好低保边缘家庭、因突发事件造成临时生活困难家庭、低保家庭大学新生入学救助工作。全面取消城乡医疗救助起付线，提高封顶线。

　　加快发展各项社会事业。一是优先发展教育事业。继续增加教育投入，优化教育结构，推动教育均衡协调发展。积极发展学前教育，加快公办中心幼儿园建设，进一步规范社会力量办园。完善提高中小学教育，加快实施中小学校舍安全工程、标准化学校建设工程和农村中小学“211”工程，加强城区中小学建设，推进城乡教育资源整合，强化素质教育，提高办学质量。整合发展职业教育，推进市、县中等职业教育资源整合，加快建设聊城高级财经职业学校和高级工程职业学校新校区。积极发展特殊教育，市区两级各建一处标准化特殊教育学校。继续大力支持聊城大学、聊城职业技术学院、东昌学院、山东工程技师学院的发展。二是促进文化大发展、大繁荣。抓好中国第十届艺术节比赛展览场馆和市图书馆的规划建设，加快县（市、区）文化馆、图书馆建设和改造，继续推进博物馆、纪念馆免费开放。加强艺术创作，继续开展送戏、送综艺、送电影下乡活动。加强文化遗产保护和管理利用。三是加快发展卫生事业。加大农民健康服务行动实施力度，加快推进公立医院改革，增加临清、茌平、高唐实施基本药物制度，稳步提高新型农村合作医疗筹资水平，进一步完善传染病预防控制体系。四是全面发展其他各项社会性事业。坚持计划生育基本国策，稳定低生育水平，优化人口出生结构。积极发展体育事业，深化体教结合，努力提升综合竞技水平；积极开展龙舟、轮滑等具有地方特色的群众体育活动，打造聊城体育亮点。健全完善双拥优抚安置服务保障体系，确保全国双拥模范城三连冠。继续加强养老服务、儿童福利、减灾救灾、残疾人、慈善等项建设，做好社会科学、民族宗教、外事侨务、对台、油区、人防、档案、史志、气象、测绘、防震等项工作，促进社会事业全面发展。

　　全力维护社会稳定。大力开展法律进机关、进学校、进企业、进村庄、进社区活动，努力营造浓厚的法治氛围。坚持主要领导定期接访制度，努力帮助群众解决生产生活中遇到的自身解决不了的困难和问题，预防和减少群体性事件的发生。以危险化学品生产企业和道路交通为重点，深入开展安全标准化建设，强化基层基础工作，推进安全生产“全员培训工程”，全力遏制较大以上安全事故的发生。进一步规范应急处置程序，提高突发事件应急处置能力。深入开展食品药品安全专项整治，全面提高安全保障水平。加强社会治安综合治理，完善社会治安防控体系，依法严厉打击各类违法犯罪活动，努力维护社会和谐稳定。

　　四、进一步加强政府自身建设

　　各位代表，实施好“十二五”规划，全面完成今年经济社会发展的各项任务，对政府工作提出了新的更高要求。面对新形势、新任务，我们有决心、有信心，把政府工作做得更加扎实、更加富有成效，进一步开创政府工作的新局面。

（一）行政效能要明显提升。要以转变政府职能为前提，以提高行政许可服务水平为重点，以加强政府目标责任管理为保障，推动政府工作全面提速。要切实加快政府职能转变，进一步精减、下放行政许可事项，最大限度地发挥市场在资源配置中的基础性作用。加强市、县两级行政服务中心建设，完善项目联审联办制度，努力缩短审批时间，提高审批效率。积极推进政务信息化，提高网上办事和服务水平。进一步完善政府目标责任管理，对每项工作都要做到目标明确、责任到人、严格考核、兑现奖惩，充分调动积极性和主观能动性，为提升行政效能提供强有力的制度保障。

　　（二）工作作风要明显改进。坚持不懈地加强作风建设，着力提高开拓创新能力和为民服务水平。一是深入调研，勇于创新。要进一步完善学习制度，广泛开展调查研究，做好上级政策与本地本部门实际结合的文章，创新工作思路、工作方式、工作方法，努力开创各项工作的新局面。二是知难而进，敢于争先。各级各部门的工作，不仅要纵向比，更要横向比，比在全省的位次，比在全省所占的比重。要敢于攻坚、敢于争先，集中力量使工作一项一项实现超越，最终实现聊城的跨越发展。三是扎实苦干，敢于担当。要坚决克服“等、靠、要”思想，按照责权利相一致的原则，明确分工，强化责任，狠抓落实。各级干部在自己的职权范围内，要大胆工作，敢于克服困难，敢于化解矛盾，敢于承担责任，杜绝“矛盾上交”行为。四是联系群众，勤政为民。我们的政府是人民的政府，考虑问题、研究工作都要把群众利益放在首位，把保障和改善民生作为出发点和落脚点。要密切联系群众，从群众实际需要出发想事情、干工作，切实为群众排忧解难，让广大群众在改革发展中得到更多实惠。

　　（三）法治水平要明显提高。要以提高政府公信力和执行力为目标，严格按照法律法规开展工作，加快法治政府建设。坚持依法科学民主决策，进一步规范决策程序，完善评估机制和责任追究制度。坚持依法统计，提高统计数据质量。完善行政执法体制，合理界定执法权限，明确执法责任，推进综合执法。规范行政执法行为，建立完善行政裁量权基准制度，加强执法评议考核。要主动接受人大及其常委会的工作监督、法律监督和政协的民主监督，高度重视新闻舆论监督和社会公众监督。全面推进政务公开，以与人民群众利益密切相关的公共部门和容易滋生腐败的领域为重点，扩大政务公开的渠道和范围，增强政府工作的透明度。认真办好市长公开电话，扎实开展行风评议、社情民意调查，畅通群众诉求渠道。

　　（四）廉政建设要明显加强。坚持预防和惩治并举，切实加强党风廉政建设，努力打造廉洁政府。认真贯彻落实《党员领导干部廉洁从政若干准则》和党政领导干部选拔任用工作四项监督制度，管好自己，管好家属子女，管好身边的同志，做廉洁自律的表率。坚持“一岗双责”，认真落实领导干部党风廉政责任制，切实抓好惩治和预防腐败体系建设。加强审计工作，加快六大电子监察平台建设，强化对决策权、执行权的监督和制约。加大案件查处力度，严肃查处重点领域和关键岗位的腐败案件，加大对群众反映强烈的突出问题的治理，树立良好的政府形象。

　　“雄关漫道真如铁，而今迈步从头越”。各位代表，站在新起点，再创新优势，实现新跨越，是全市人民的热切期盼，是时代赋予我们的光荣使命。我们要更加紧密地团结在以胡锦涛同志为总书记的党中央周围，凝聚全市人民的智慧和力量，坚定信心，锐意进取，扎实苦干，推动经济长期平稳较快发展和社会和谐稳定，在建设生态型强市名城的道路上迈出新的更大的步伐！
